# Osseous microarchitecture in frequent fracture zones of the distal clavicle

**DOI:** 10.1016/j.jseint.2022.09.015

**Published:** 2022-10-20

**Authors:** Jan Gewiess, Christoph Sprecher, Stefan Milz, Johannes Gleich, Tobias Helfen

**Affiliations:** a Department of Orthopaedic Surgery and Traumatology, Inselspital, Bern University Hospital, University of Bern, Bern, Switzerland; b AO Research Institute Davos, Davos, Switzerland; c Department of Anatomy II, Ludwig-Maximilians University, Munich, Germany; d Shoulder and Elbow Service, Department of General, Trauma and Reconstructive Surgery, Munich University Hospitals, Ludwig-Maximilians-University, Munich, Germany

**Keywords:** Distal clavicle, Fracture, Bone quality, Osteoporosis, XtremeCT, Bone mineral density

## Abstract

**Background:**

Fracture classifications of the distal clavicle are based on ligamentous integrity. The influence of osseous microarchitecture on fracture occurrence, morphology, and the lesion’s stability has not yet been investigated. We aimed to characterize osseous microarchitecture according to common fracture classification systems based on ligamentous integrity and investigated the possible effects of age, gender, and osteoporosis in distal clavicle fractures.

**Methods:**

N = 20 human cadaveric distal clavicles were scanned using XtremeCT with an isometric voxel size of 82 μm. In the sagittal plane, each data set was evaluated in 11 sections of approximately 7 mm thickness. Three topographic regions were defined: the bone lateral to the trapezoid (LTR), intertubercular (ITR), and medial to the conoid (MCR) ligament. Cortical bone mineral density (BMD) [mgHA/cm^3^] and cortical porosity (1- (BV/TV) [%]) were determined and evaluated relative to age and gender.

**Results:**

Along the mediolateral axis, there was an >20-fold increase in median cortical porosity (*P* ≤ .001). There were significant differences in cortical porosity between LTR and ITR (*P* ≤ .001) but not between ITR and MCR (*P* = .09). In ITR, cortical porosity was significantly greater in >60-year-old compared to younger donors (*P* = .01). For BMD, there was an >2-fold decrease toward the distal apex (*P* ≤ .001). BMD was significantly greater in ITR compared to LTR (*P* ≤ .001) and in MCR compared to ITR (*P* = .02). In ITR and MCR, clavicles of >60-year-old donors had significantly lower BMD values compared to younger donors (*P* < .01). Across all 3 regions, frequency distribution of low bone mass did not significantly differ between <60-year-olds and >60-year-olds (*P* > .6).

**Conclusion:**

The distal clavicle features a characteristic bony microarchitecture. The present study revealed a significant difference in bone quality of lateral, intertubercular, and medial zones of the distal clavicle and could specify target areas and strategies for surgical treatment of unstable fractures. Age, gender, and osteoporosis have a limited effect on bone quality and fracture genesis. In contrast, ligamentous quality is supposed to exert a substantial influence on fracture characteristics, especially in ITR. Fracture morphology of the distal clavicle is determined by a bony-ligamentous conjunction, which remains to be characterized.

Clavicle fractures account for almost half of all fractures in the shoulder girdle.^18^ In 10%-30%, the distal third is affected.^17^ Differential therapeutic management is indicated based on fracture stability, age, and functional demands.^1^^,^^19^

Conclusions regarding stability of distal clavicle lesions can be drawn from fracture classification systems. Common fracture classification systems of Neer,^15^ Jäger and Breitner,^9^ or Robinson^21^ illustrate the role of ligamentous integrity in determining fracture stability, following anatomical considerations of the fracture configuration in relation to the acromioclavicular (AC) and the coracoclavicular (CC) ligaments. Given the lesion’s heterogeneity, none of the existing classification systems has proven to be comprehensive.^12^ This inconsistency might reflect the so far uncharted osseous microstructure and its implications regarding the predetermination of fracture configuration and stability.

Various bones are subject to age- and gender-related remodeling processes with an associated risk of fracture. As such, the proximal humeral fracture is considered as an indicator for osteoporosis of the shoulder girdle.^24^ Similarly, distal clavicle fractures have been shown to affect different age groups with two incidental peaks^11^^,^^22^: While young and active male adults usually present with injuries resulting from a direct blow to the shoulder, there is an increasing number of distal clavicle fractures in elderly females with low bone quality. An investigation of the osseous microstructure of the distal clavicle may provide an explanation comparable to those widely accepted for proximal humeral fractures.

Using XtremeCT scans of human cadavers, this study aimed to characterize the osseous microstructure of the distal clavicle, to correlate these findings to common fracture classification systems, and to investigate the effect of age and gender on bone quality.

## Methods

### Material and parameters

**Figure 1:**
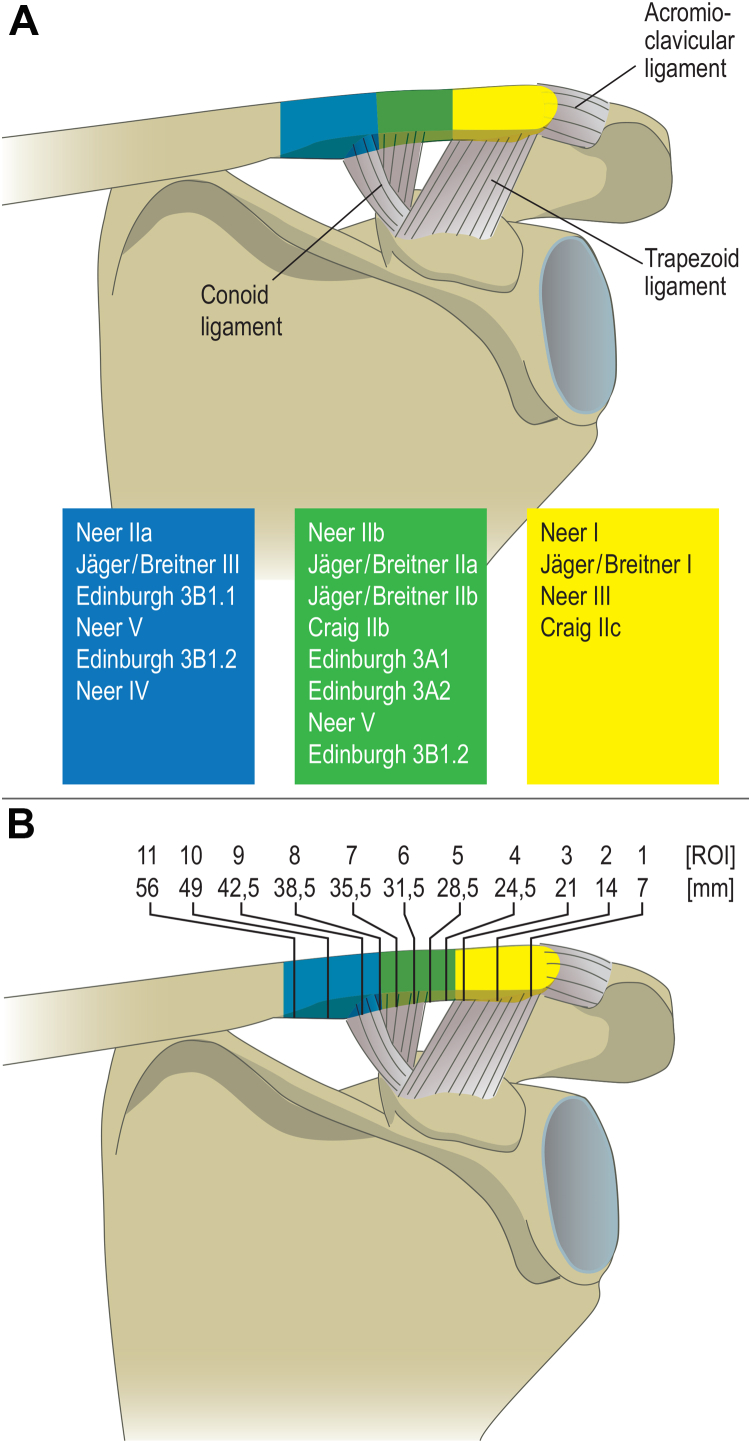
According to fracture classification systems, the distal clavicle was sectioned in regions located medial to the conoid tubercle (*blue*), intertubercular (*green*), and lateral to the trapezoid tubercle (*yellow*). **A**: Fracture regions of the distal clavicle according to common classification systems of Neer, Jäger/Breitner, Craig and Edinburgh. Fracture stability depends on concomitant ligamentous injury. Instability is assumed in Neer IIA, Jäger/Breitner III, Neer IIB, Jäger/Breitner IIA, Neer V, Edinburgh 3B and Craig IIC.^23^**B**: Scheme of ROI distribution along the mediolateral axis in relation to anatomic landmarks. We designed 4 regions of interest (ROI) medial to the conoid tubercle center (MCR), one ROI at the conoid tubercle center, two ROI within the intertubercular region (ITR), one ROI at the trapezoid tubercle center, and three ROI lateral to the trapezoid tubercle (LTR).

**Figure 2:**
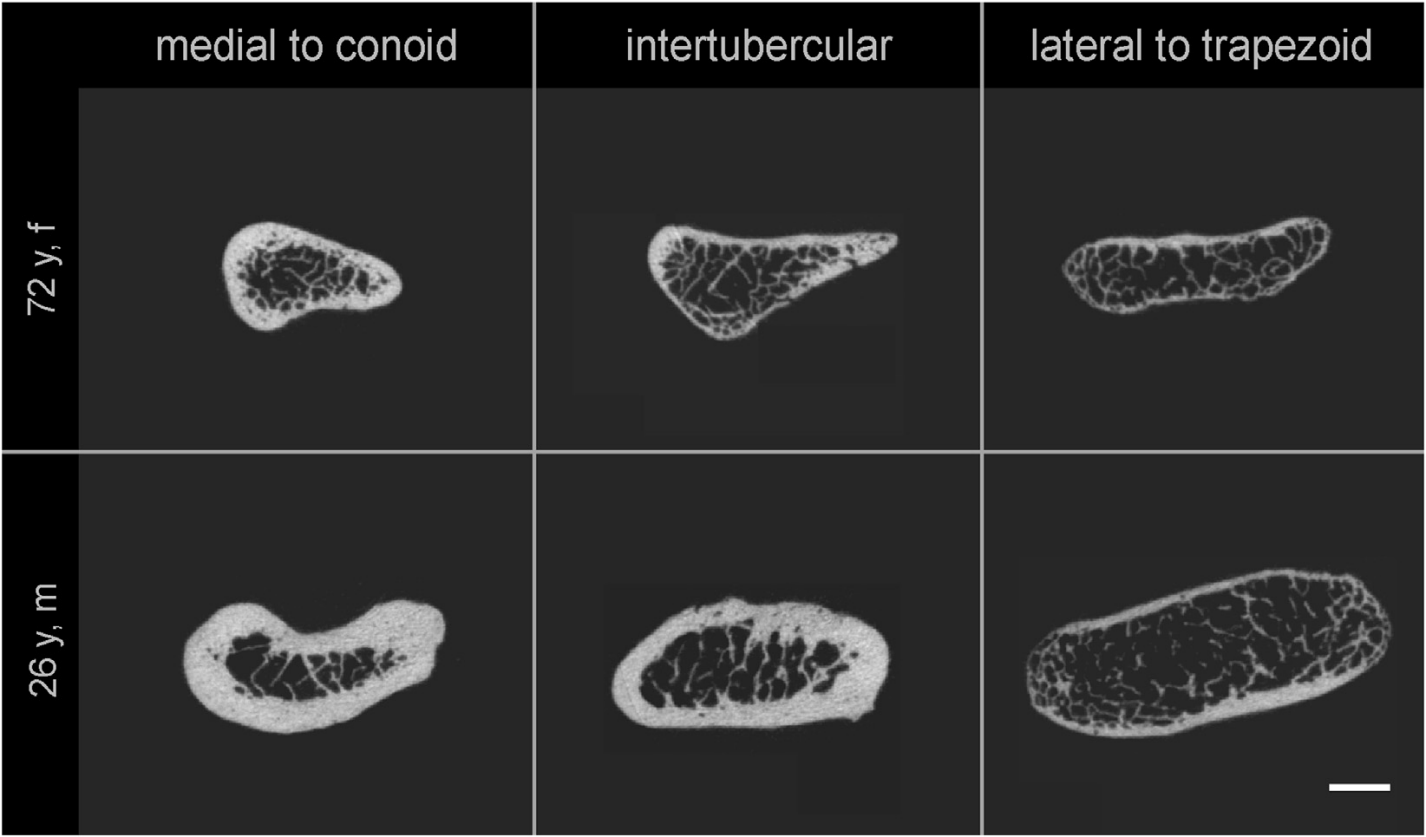
XtremeCT scans of clavicle fracture sections in representative 72-year-old female (*upper row*) and 26-year-old male (*lower row*) specimen. The clavicular cross-sectional bone morphology features high interindividual variance with a remarkable flattening and cortical thinning toward the lateral apex existent consistently regarding age and gender. Bar = 5 mm.

N = 20 (n = 10 male, n = 10 female, mean age [SD] = 57 [20.7] years) fresh frozen left human cadaveric clavicles were examined, with the soft tissue removed, leaving only the ligamentous entheses in place (Table I). Specimens were recruited from the Forensic Institute of the Ludwig-Maximilians-University (LMU) after approval by the local ethics committee (registration nr.: 499-15). CT scans with an isotropic voxel size of 82 μm were preserved using XtremeCT (XtremeCT, Scanco Medical AG, Brüttisellen, Switzerland). Normalized to each bone’s individual length, the distal thirds of the clavicles were subdivided into 11 sections at approximately 7 mm distances in the sagittal plane (Fig. 1). Centers and footprints of the CC- entheses were referenced and normalized according to Rios et al with the center of the trapezoid enthesis being located at a mean distance of 25.9 mm and the center of the conoid enthesis at a mean distance of 35 mm from the distal apex. Along the mediolateral axis, different fracture types were localized according to the following regions of interest (ROI, Fig. 1): medial to the conoid tubercle (MCR, two ROI), conoid tubercle (one ROI), intertubercular region (ITR; two ROI), trapezoid tubercle (one ROI), and lateral to the trapezoid tubercle (LTR, three ROI). Each ROI consisted of 25 consecutive images (approx. 2 mm). Across each ROI, parameters of bone quality were determined using a standardized and previously described method based on automated image segmentation using adaptive thresholding.^14^ Bone mineral density (BMD) [mgHA/cm^3^], cortical bone volume (BV)[mm³], and cortical porosity, described as 1- bone volume/total volume (1-(BV/ (TV))[%]) were evaluated.^2^ All parameters of cortical bone quality were compared between male and female, and between <60-year-old (25-59 y) and >60-year-old (60-87 y) donors (Fig. 2).

**Table I:** Overview of clavicle donors.

ID	Age [y]	Sex	Clavicle length [mm]	Mean BMD [mgHA/cm^3^]	Median cortical porosity [%]
25-59 y					
1	44	m	180	909.2	1.5
2	26	m	100	882.6	1.5
3	45	m	120	804.5	3.6
4	25	f	80	856.6	0.7
5	29	f	85	941.5	0.8
6	37	f	80	709.3	4.5
7	36	f	145	761.2	4.1
8	40	f	145	1008.3	0.9
central tendency	35 (7.8)∗		117 (37)∗	859.2 (97.9)∗	1.5 (2.2)†
60-87 y					
9	72	f	165	761.0	2.2
10	78	m	170	800.4	3.2
11	62	m	180	833.0	3.0
12	82	m	170	797.1	2.4
13	86	m	190	704.1	5.2
14	67	m	185	796.1	2.0
15	87	f	90	722.5	4.7
16	64	m	90	733.9	10.0
17	81	f	90	555.9	7.7
18	66	f	100	921.4	1.7
19	64	m	110	800.1	2.4
20	64	f	120	762.2	2.2
central tendency	73 (9.5)∗		138 (42)∗	765.6 (87.3)∗	2.7 (2.6)†

*BMD*, bone mineral density.

∗ Mean (standard deviation).

† Median (interquartile range).

According to the World Health Organization (WHO), osteoporosis is defined by a BMD of 2.5 standard deviation (SD) or more below the young adult mean of 30-year-old, healthy individuals. In this cohort, the age group of <60-year-olds had a mean age of 35.3 ± 7.5 years and the age group of >60-year-olds had a mean age of 72.8 ± 9.2 years. Following the WHO definition, BMD values of <60-year-olds were used to define ‘osteoporosis’ and ‘low bone mass’ to demonstrate the occurrence of these in the distal clavicle.

### Statistical analysis

Statistical evaluation was performed using ‘R’ (R Core Team, Version 4.1.0; R Foundation for Statistical Computing, Vienna, Austria) and GraphPad Prism (Version 9.3.1 (2022); GraphPad Software, La Jolla, CA, USA). For determination of the minimal sample size required, an a priori power analysis was conducted using previously published BMD values of the distal clavicle,^4^ which lie within the range of 800 ± 38 mgHA/cm^3^. Considering the WHO definition with osteoporosis being present at −2.5 SDs, the required sample size to achieve 80% power for detecting this effect, at a significance criterion of α = 0.05, was n = 3 per group for an independent samples t-test.

Normal distribution was tested using the Shapiro-Wilk test. Data description was performed using mean and SD for parametric and median and interquartile range (IQR; Q3-Q1) for nonparametric data. Correlation analyses were performed using two-sided Pearson’s and Spearman’s methods, respectively. Frequency comparisons were performed using chi-squared and Fisher’s exact test for expected frequencies <5. Unpaired group comparisons were performed using t-test for parametric and Mann-Whitney U-test for nonparametric data. Pairwise comparisons were performed using paired t-tests for parametric and Wilcoxon matched-pairs signed rank test for nonparametric data. Unpaired multiple comparisons were performed using one-way ANOVA with Tukey’s correction for parametric data and Kruskal-Wallis test with Dunn’s correction for nonparametric data. Paired multiple comparison tests were performed using mixed-effects analysis with Tukey’s correction for parametric and Friedman test with Dunn’s correction for nonparametric data. The level of significance was set at *P* = .05.

## Results

### Demographic and specimen characterization

The mean age of donors was 57.8 (25-87) in females and 61.8 (26-86) in males (Table I; *P* = .4). Median overall clavicle length was 120 mm (min = 90 mm; max = 190 mm) and median length of the distal third was 56 mm (min = 5.3 mm; max = 6.7 mm). Male specimens were longer compared to female specimens (*P* = .01). Lengths of the distal thirds were comparable between gender groups (*P* = .68).

### Cortical porosity

There were significant differences between LTR and ITR (median 9.7%, IQR = 7.7 and 2.9%, IQR = 2.2; *P* < .001) but not between ITR and MCR (2.9%, IQR = 2.2 and 1.9%, IQR = 1.6; *P* > .9; Table II). In MCR, there was a trend toward greater cortical porosity in male specimen (*P* = .068) and >60-year-old donors (*P* = .0548). There was no significant gender-related difference in ITR (*P* = .753). In ITR, cortical porosity was significantly greater in >60-year-old compared to younger donors (2.8%, IQR = 2 and 1.3%, IQR = 2.3; *P* = .01).

**Table II:** Medians of cortical porosity (1-(BV/TV) [%]) values across investigated anatomical regions and ROI.

Region	LTR			TT	ITR		CT	MCR		
ROI A	1	2	3	4	5	6	7	8	9	10
	27.4%	12.9%	4.4%	3.6%	3.3%	3.5%	4.20%	3%	3.6%	1.5%
ROI B	2	3	4	5	6	7	8	9	10	11
	12.9%	4.4%	3.6%	3.3%	3.5%	4.2%	3%	3.6%	1.5%	1.3%

*CT*, conoid tubercle; *ITR*, intertubercular region; *LTR*, lateral to trapezoid; *MCR*, medial to conoid; *ROI*, region of interest; *TT*, trapezoid tubercle.

There were no significant age-related or gender-related differences in LTR (*P* = .2026 and *P* = .997). However, across all 3 regions, there were no significant differences between young female, young male, old female, and old male donors regarding cortical porosity (*P* > .0761).

### Bone mineral density (BMD)

**Figure 3:**
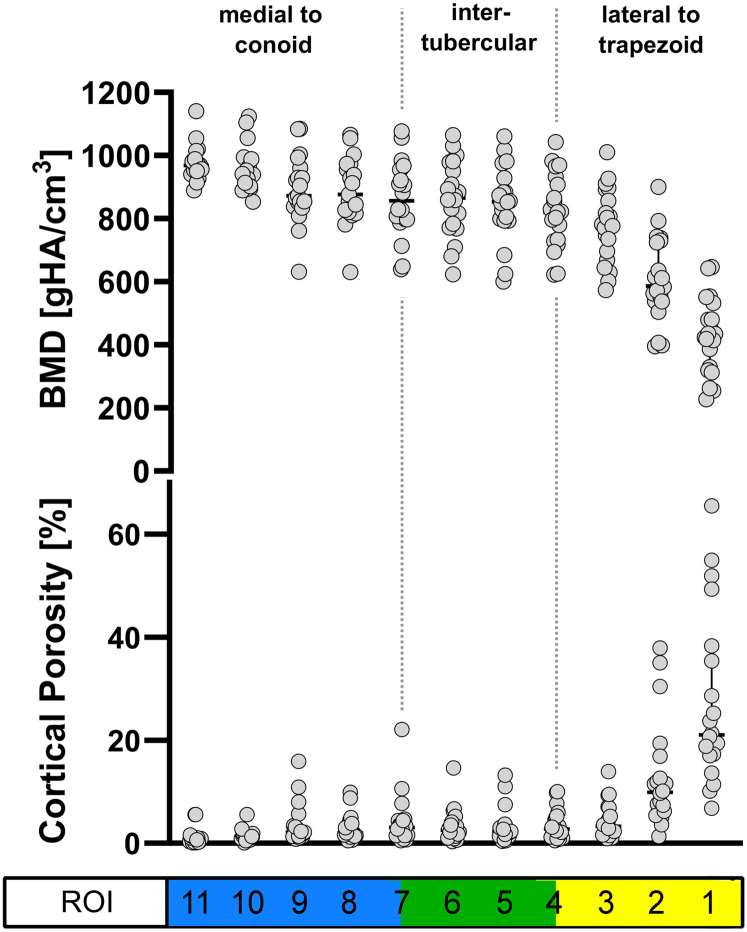
Cortical porosity [%] and cortical BMD [mgHA/cm^3^] along the mediolateral axis of the clavicle. Fracture regions are displayed according to common fracture classification systems. There was a significant, >20-fold increase in cortical porosity toward the lateral apex. There was a significant, >2-fold decrease in BMD toward the lateral apex. Cortical porosity: bar = median; whiskers = IQR; BMD: bar = mean; whiskers = SD. *BMD*, bone mineral density; *SD*, standard deviation; *IQR*, interquartile range.

Mean cortical BMD in the distal third of all clavicles was 806.8 mgHA/cm^3^. There was an >2-fold decrease toward the distal apex (mean BMD ROI 1 = 427.6 ± 120.5 mgHA/cm^3^ and ROI 11 = 974.7 ± 58.8 mgHA/cm^3^; *P* < .001) (Table III, Fig. 3). BMD was significantly greater in ITR compared to LTR (858.5 ± 118.9 mgHA/cm^3^ vs. 603.9 ± 188.9 mgHA/cm^3^; *P* < .001) and in MCR compared to ITR (905.7 ± 100.8 mgHA/cm^3^ vs. 858.5 ± 118.9 mgHA/cm^3^; *P* = .042) (Fig. 3).

**Table III:** Means of BMD [mgHA/cm^3^] values across investigated anatomical regions and ROI.

Region	LTR			TT	ITR		CT	MCR		
ROI A	1	2	3	4	5	6	7	8	9	10
	427.6 ± 120.5	606.7 ± 134.1	777.3 ± 118.0	824.8 ± 117.9	853.6 ± 122.1	863.5 ± 118.6	863.4 ± 120.8	889.8 ± 105.2	894.0 ± 107.5	955.0 ± 76.9
ROI B	2	3	4	5	6	7	8	9	10	11
	606.7 ± 134.1	777.3 ± 118.0	824.8 ± 117.9	853.6 ± 122.1	863.5 ± 118.6	863.4 ± 120.8	889.8 ± 105.2	894.0 ± 107.5	955.0 ± 76.9	974.7 ± 58.8

*BMD*, bone mineral density; *CT*, conoid tubercle; *ITR*, intertubercular region; *LTR*, lateral to trapezoid; *MCR*, medial to conoid; *ROI*, region of interest; *TT*, trapezoid tubercle.

In LTR, there was no significant difference in BMD of <60-year-old and >60-year-old donors (652.5 ± 207.2 mgHA/cm^3^ and 571.5 ± 171 mgHA/cm^3^; *P* = .1). In ITR and MCR, clavicles of <60-year-old donors had significantly greater BMD values compared >60-year-old donors (ITR: 914.8 ± 133.9 mgHA/cm^3^ and 821 ± 92.8 mgHA/cm^3^; MCR: 959 ± 99.9 mgHA/cm^3^ and 877 ± 87.8 mgHA/cm^3^; *P* < .01).

Mean BMD was lower in clavicles of female donors across the distal third, but significant gender-related differences could not be established (LTR, male: 616. 2 ± 154.8 mgHA/cm^3^; female: 591.5 ± 219.8 mgHA/cm^3^; ITR, male: 866 ± 77.5 mgHA/cm^3^; female: 851 ± 151.3 mgHA/cm^3^; MCR, male: 912 ± 49.8 mgHA/cm^3^; female: 910.7 ± 134 mgHA/cm^3^; *P* > .6).

BMD was greater across all 3 regions in <60-year-old compared to >60-year-old female donors, but significance was present in MCR only (LTR: 464.8 ± 147.5 mgHA/cm^3^ and 314.8 ± 79.1 mgHA/cm^3^; *P* = .343; ITR: 890.5 ± 191.7 mgHA/cm^3^ and 804 ± 124.6 mgHA/cm^3^; *P* = .1871; MCR: 67 ± 124.5 mgHA/cm^3^ and 854.4 ± 122.2 mgHA/cm^3^; *P* = .012). Clavicles of the old female group consistently had the lowest BMD values regarding fracture zone, however in ITR and LTR, there were no significant differences in any of the groups (young female, young male, old female, and old male; *P* > .187).

**Figure 4:**
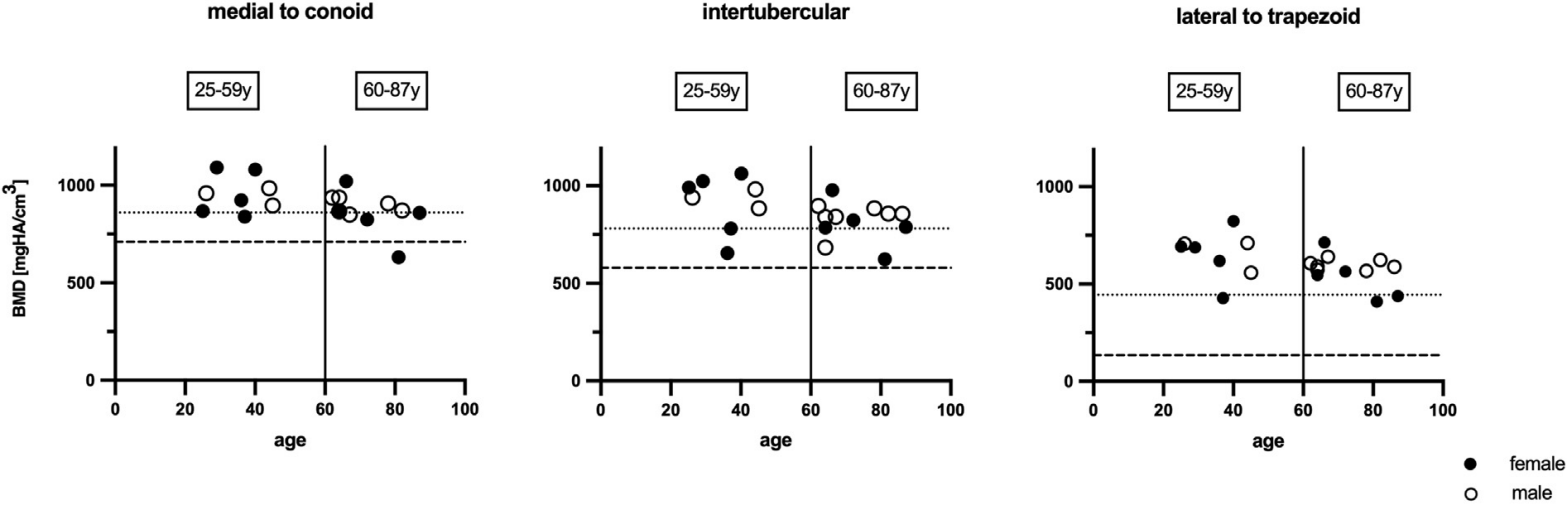
BMD and age in fracture zones of the distal clavicle. According to the World Health Organization, osteoporosis is defined by a BMD of 2.5 SD or more below the young adult mean of 30-year-old, healthy individuals. In this cohort, the age group of <60-year-olds had a mean age of 35.3 ± 7.5 years and the age group of >60-year-olds had a mean age of 72.8 ± 9.2 years. Mean BMD (SD) for <60-year-olds were 959.2 (99.9) mgHA/cm^3^ medial to the conoid, 914.8 (133.9) mgHA/cm^3^ in the intertubercular region, and 652.5 (207.2) mgHA/cm^3^ lateral to the trapezoid. BMD values of both, female and male specimen were altered with age in a similar fashion. Frequencies of low bone mass (MCR: 12.5% and 33.3%; ITR and LTR: 12.5% and 16.7%) and osteoporosis (MCR: 0% and 8.3%; ITR and LTR: 0% in both groups) did not differ between both age groups in the respective fracture region (*P* > .6). Dotted line = −1 SD of 25-59 y (threshold of low bone mass). Dashed line = −2.5 SD of 25-59 y (threshold of osteoporosis). *BMD*, bone mineral density; *SD*, standard deviation; *MCR*, medial to conoid; *ITR*, intertubercular region; *LTR*, lateral to trapezoid.

Correlation analysis showed a trend toward consistently reduced BMD with increasing age across all 3 regions but did not reach statistical significance. Across the distal third, frequency distribution of osteoporosis (0% in both groups) and low bone mass (0% 25-59 y and 8.3% in 60-87 y) did not significantly differ between age groups (*P* > .99). When adjusting for the respective fracture region, no further significant difference was established (Fig. 4).

## Discussion

Common systems for distal clavicle fracture classification according to Neer,^15^ Jäger and Breitner,^9^ Robinson or Craig^21^ are geared to the fracture’s localization in relation to the concomitant integrity of the CC ligaments. To evaluate the influence of bony microstructure on distal clavicle fracture occurrence in its ligamentous context, the present study evaluated XtremeCT scans of human cadaveric distal clavicles.

Specimens were representatively selected regarding age and gender distribution. Demographic data did not significantly differ between investigated groups. Methodological considerations such as age group definitions,^10^^,^^24^ parameters regarding length and localization of ligamentous enthesis areas^6^^,^^8^ as well as the use of XtremeCT and the modality of fractionation^4^^,^^7^ for the evaluation of our hypothesis have been approved regarding eligibility in previous studies.

According to ligamentous and bony topography, we defined 3 different sections within the distal clavicle. Cortical porosity continuously increased from the medial to the lateral section.

A highly significant increase in cortical porosity could be shown especially for the most lateral section. Here, fracture types Neer I (Jäger/ Breitner I), Neer III, and Craig IIc are located. While Neer I (Jäger/ Breitner I) and Neer III fractures represent stable fractures, Craig IIc fractures are considered unstable.^17^ Assumptions on stability are derived from ligamentous integrity. Despite the nonexistence of comparative epidemiological data, our results suggest an ancillary influence of bony microstructural processes on injury pattern within the lateral distal clavicle section. Consequently, the Craig IIc injury pattern might be related to compromised quality or aberrant course of stabilizing ligaments,^3^^,^^13^ marked changes to their entheses^16^ or the mechanistic scenario and acting force vectors.^23^

Distinct age- and gender-related differences in cortical porosity were particularly observed in the transitional zone toward the diaphysis. The authors attributed these changes to common bony remodeling aspects rather than osteoporotic transformation. The observed significant age-related increase in cortical porosity in the intertubercular region is liable to a similar interpretation.

Corresponding to cortical porosity, BMD was found to decrease from the medial toward the lateral section of the distal clavicle. Differences were significant regarding all 3 defined sections of the distal clavicle. Based on this observation, most fracture types would be expected within the section borders, if bony influence was clearly relevant. However, regarding the above-mentioned classification systems, only half of all fractures feature a respective localization (Neer I, Neer III, Neer IIa, Craig IIc). The remainder is localized within the intertubercular region (Jäger/ Breitner IIb, Edinburgh 3A1 & 3A2, Neer IIb, Neer V). Again, fracture genesis cannot be sufficiently explained by bone quality in general, and BMD in particular, and rather has to be interpreted considering an interplay of course and quality of surrounding ligaments, entheses and acting force vectors.

Concordantly to the observed conditions regarding cortical porosity of the most lateral section, there was a comparably lower BMD of younger male specimens. Besides an argumentation regarding skeletal maturation, higher prevalence of arthritis of the AC joint in the elderly may provide further explanation. A clinically relevant contribution of osteoporosis as deducted in the present study in distal clavicle fracture genesis can be considered virtually nonexistent: An analysis using cohort specific results of the present study yields an estimated power value of 1.

Regarding both parameters, cortical porosity and BMD, bone morphology is characteristic but can only partially be considered to exert an influential effect on the occurrence and stability of distal clavicle fractures. Thus, an in-depth imaging, histological, and biomechanical analysis of the CC ligaments, their entheses, and the AC joint including the surrounding capsule during the fracture process may provide further clarification.

Within the intertubercular region, some uncertainty remains regarding the bony-ligamentous ratio and its influence on fracture occurrence. However, compared to the lateral section, a significantly better bone quality with respect to osteosynthesis was shown in the present study. The area of central insertion of the CC ligaments at the clavicle includes approximately 20.1 mm in females and 22.3 mm in males.^20^ Thus, this section is relatively broad and bone stock for osteosynthesis varies depending on fracture localization. Importantly, age and gender turned out not to be reliable predictors of bone quality. For unstable fractures of this region (Neer IIb, Jäger/ Breitner IIa, Neer V, unstable Edinburgh 3B1), the authors recommend techniques, which allow sufficient neutralization of cranialization forces exerted by dynamic stabilizers, for example, plate osteosynthesis, which may be augmented by CC stabilization. In this situation, any attempt of neutralization by the use of isolated CC stabilization techniques seems to be associated undertreatment.^5^

This study has several limitations including the absence of health records of respective donors. Unequal gender distributions complicated conclusions regarding age-related alternations of BMD. However, this effect was equally present in female and male specimens. Thus, the presented approach to identify low bone stock or osteoporosis can only be approximative. Image analysis with automated and autonomic segmentation using adaptive thresholding, though eliminating the subjective impact of interpretation, theoretically risks preclusion of user-controlled visualized correction. The suggested approach of defining cortical porosity based on cortical bone volume fraction cannot meet histological precision as the partial volume effect must be considered to preclude identification of even the smallest vascular and resorption cavities. Otherwise, this shortcoming might be limited at least partially by its consistency across all measurements. Importantly, and given the heterogenous morphology of the clavicle, an exact definition of the location of ligamentous entheses and the intertubercular region from XtremeCT scans is not possible. Besides epidemiologic fracture characterization, future research might engage imaging, histological and biomechanical methods in order to mimic and understand fracture genesis in light of exact anatomic relations.

## Conclusion

The distal clavicle features a characteristic bony microarchitecture. The present study revealed a significant difference in bone quality of lateral, intertubercular, and medial regions of the distal clavicle and could specify target areas and strategies for surgical treatment of unstable fractures. Age, gender, and osteoporosis have a limited effect on bone quality and fracture genesis. In contrast, ligamentous quality is supposed to exert a substantial influence on fracture characteristics, especially in ITR. Fracture morphology of the distal clavicle is determined by a bony-ligamentous conjunction, which remains to be characterized. Further studies on ligaments are necessary.

## Disclaimers

Funding: Research was supported by funds from the FöFoLe program of the Medical Faculty of the 10.13039/501100005722LMU, Munich, Germany.

Conflicts of interest: The authors, their immediate families, and any research foundation with which they are affiliated have not received any financial payments or other benefits from any commercial entity related to the subject of this article.
